# Transcriptomic insights into the interplay between polyketide biosynthesis and other secondary metabolite biosynthetic clusters and biological pathways in entomopathogen *Beauveria bassiana*

**DOI:** 10.3389/fmicb.2025.1583637

**Published:** 2025-05-23

**Authors:** Wachiraporn Toopaang, Thippawan Yoocha, Chaiwat Naktang, Nuchnudda Wichienchote, Phornsiri Pechsrichuang, Sithichoke Tangphatsornruang, Morakot Tanticharoen, Yu-Liang Yang, Alongkorn Amnuaykanjanasin

**Affiliations:** ^1^National Center for Genetic Engineering and Biotechnology, National Science and Technology Development Agency, Pathum Thani, Thailand; ^2^Molecular and Biological Agricultural Sciences Program, Taiwan International Graduate Program, Academia Sinica and National Chung Hsing University, Taipei, Taiwan; ^3^Agricultural Biotechnology Research Center, Academia Sinica, Taipei, Taiwan; ^4^Graduate Institute of Biotechnology, National Chung Hsing University, Taichung, Taiwan; ^5^National Omics Center, National Science and Technology Development Agency (NSTDA), Pathum Thani, Thailand; ^6^School of Bioresources and Technology, King Mongkut’s University of Technology Thonburi, Bangkok, Thailand; ^7^Biotechnology Center in Southern Taiwan, Academia Sinica, Tainan, Taiwan

**Keywords:** *Beauveria bassiana*, polyketide synthase, transcriptome, secondary metabolite, crosstalk

## Abstract

**Background and aims:**

The polyketide synthase gene *pks15* plays a critical role in insect virulence and cell wall formation in the entomopathogenic fungus *Beauveria bassiana*. Metabolomics studies have also shown that this gene exhibits crosstalk with other biosynthetic clusters of beauvericins, bassianolide, enniatin A, and ferricrocin. Here, we investigated the cross-pathway communication of *pks15* biosynthetic cluster and other secondary metabolite clusters and biological pathways using transcriptomes.

**Methods:**

Two comparative transcriptomic analyses were conducted, one compared the wild-type *B. bassiana*-injected beet armyworm (WT *in vivo*) with Δ*pks15* mutant-injected beet armyworm (Δ*pks15 in vivo*), and the other one compared WT *in vivo* with wild-type grown *in vitro*. Insect inoculation was performed by intrahemocoelic injection of conidia, hence bypassing the cuticular penetration.

**Results:**

The transcriptomic profile of Δ*pks15 in vivo* revealed significant downregulation of genes involved in mycotoxin production, secondary metabolite biosynthesis, and cell wall integrity compared to the WT *in vivo*. Notably, 36 out of 45 secondary metabolite biosynthetic clusters in *B. bassiana* BCC 2660, were downregulated in Δ*pks15 in vivo*, suggesting marked changes in the biosynthesis of secondary metabolites after *pks15* deletion. These clusters included genes encoding nonribosomal peptide synthetase, transporters, glycosylation, proteolysis, peptidase activity, signal peptides, and cell wall and surface proteins. Our findings indicate that *pks15* plays an important role in fungal development and pathogenicity. Within the *pks15* cluster, the UDP-glucosyl transferase gene *Bbugt1* was consistently upregulated 3-fold in the WT *in vivo* compared to the WT *in vitro* armyworm group 48–96 h post-inoculation. In contrast, *Bbugt1* was downregulated in Δ*pks15 in vivo* compared to the WT *in vivo* during the same period. This regulation pattern suggests that *Bbutg1* plays a role in the production or modification of secondary metabolites, specifically during the host infection.

**Conclusion:**

This study provides the first transcriptomic evidence that the *pks15* cluster regulates multiple secondary metabolite clusters, including bassianolide, siderophores, tenellin, oosporein, and several unidentified PKS and NRPS clusters. Additionally, *pks15* is associated with fungal cell wall remodeling and immune evasion. Our work uncovers an expanded regulatory role for PKS15, revealing novel connection between metabolite biosynthesis and virulence-associated processes, and offering opportunities for targets for biocontrol improvement and metabolite engineering.

## 1 Introduction

*Beauveria bassiana* is a well-known entomopathogenic fungus widely utilised in biological control. It is used to manage various insect pests such as diamondback moth, European corn borer, corn earworm, beet armyworm, cabbage looper, and fall armyworm (Wraight et al., 2010). Major secondary metabolites produced by this fungus are polyketides, nonribosomal peptides (NRPs), and hybrid polyketide-NRPs, which play significant roles in both medicine and agriculture. Well-known NRPs, including beauvericins, bassianolide, and ferricrocin, serve as virulence factors in *B. bassiana* (Eisendle et al., 2006; Hof et al., 2007; Sy-Cordero et al., 2012; Wang and Xu, 2012; Yu et al., 2013). In addition to polyketides, NRPs, and hybrid polyketide-NRPs, ribosomally synthesized and post-translationally modified peptides (RiPPs) are also classified as secondary metabolites in bacteria and fungi. These RiPPs exhibit diverse bioactivities, including antimicrobial activities (Schnell et al., 1988), quorum-sensing (Stock et al., 2000), the formation of aerial hyphae and spore formation (Kodani et al., 2005), antiviral activities (Meindl et al., 2010), toxic virulence factors, and signaling molecules that induce morphological changes and secondary metabolite production (Onaka et al., 2001). Polyketide synthases (PKSs), which synthesize polyketides, are abundant in entomopathogenic fungi, for example, *Metarhizium robertsii* (ARSEF 2575) carrying 24 PKS genes, *M. acridum* carrying 13 PKS genes, *B. bassiana* carrying 12 PKS genes (Gao et al., 2011; Punya et al., 2015). However, to date, polyketides have rarely been identified in entomopathogenic fungi. *B. bassiana* BCC 2660 has 12 PKS genes. Two PKS genes, *pks14* and *pks15*, are highly conserved in entomopathogenic fungi (Amnuaykanjanasin et al., 2009; Punya et al., 2015).

Our previous studies demonstrated that *pks15* plays a crucial role in fungal development, virulence, and cell wall organization in *B. bassiana*. Specifically, the Δ*pks15* mutant exhibited notable phenotypic defects, including reduced conidiation, reduced spore germination, and attenuated insect virulence, all of which can be restored by genetic complementation (Toopaang et al., 2017; Udompaisarn et al., 2020). Moreover, PKS15 contributes to immune evasion, as shown by the reduced phagocytic survival of Δ*pks15* mutant blastospores in *Acanthamoeba castellanii* compared to *B. bassiana* wild type. Cell structural analyses further revealed that deficiency of PKS15 results in altered conidial morphology, including the absence of rodlet bundles on the cell wall surface and changes in mannan/glucan accessibility, suggesting that PKS15 influences both the structural and biochemical integrity of the fungal surface (Toopaang et al., 2017; Udompaisarn et al., 2020). More recent metabolomic analyses have further implicated PKS15 in regulating the biosynthesis of several NRPs and siderophores, including beauvericins, bassianolide, enniatin A, and ferricrocin during the host infection (Toopaang et al., 2023). These results suggest that PKS15 may serve not only as a biosynthetic enzyme but also as a cross-pathway regulator of other secondary metabolite biosynthetic gene clusters (BGCs). However, this regulatory role remains unexplored. Crosstalk between distinct secondary metabolite BGCs has been widely demonstrated in fungi, allowing the production of complex chemical scaffolds through coordinated regulation. For example, the synthesis of penilactones A and B in *Penicillium crustosum* (Fan et al., 2019), dalmanol A and acetodalmanol A in *Daldinia eschscholzii* (Zhang et al., 2008; Zhang et al., 2011; Zhang et al., 2016), and asperfuranone in *Aspergillus nidulans* (Bergmann et al., 2010) exhibited the functional connectivity between polyketide and nonribosomal peptide biosynthetic clusters. These secondary metabolites had various biological activities such as immunosuppressive, anticancer, and cell cycle—inhibiting properties (Wu et al., 2012; Dai et al., 2022; Hu et al., 2024). Nevertheless, knowledge about how PKS15 influences fungal development and facilitates fungal pathogenicity remains limited. Investigating the crosstalk between gene clusters will reveal novel compounds and diverse bioactivities, paving the way for the discovery of potential secondary metabolites.

Integrating transcriptomics with metabolomics reveals the correlation between gene expression and metabolite accumulation (Zhao et al., 2022). Transcriptome analysis helps in identifying uncharacterized genes involved in novel metabolic pathways (Cavicchioli et al., 2022), mapping cross-relationships (Hladik et al., 2022), and exploring the relationship between pathological processes and their corresponding metabolic pathways, facilitating the identification of potential biomarkers and therapeutic targets relevant to specific stress conditions (Dutta et al., 2020; Ma et al., 2021; Maan et al., 2023). However, the application of transcriptomics to investigate cross-relationships in the secondary metabolite biosynthesis in entomopathogenic fungi remains limited.

In this study, we addressed this limit by conducting a comparative transcriptomic analysis of *B. bassiana* wild type and the Δ*pks15* mutant during insect infection. We found that several secondary metabolite BGCs were significantly downregulated in the Δ*pks15* mutant. These included genes encoding nonribosomal peptide synthetases (NRPS), metabolite transporters, and cell wall structure, indicating a global regulatory role for PKS15. To our knowledge, this is the first comprehensive transcriptomic evidence that PKS15 or its associated polyketide orchestrates cross-cluster regulation in modulating the expression of multiple BGCs in *B. bassiana*. This regulation influences secondary metabolite production, virulence-associated pathways, and cell wall remodeling in *B. bassiana*, thereby coordinating diverse biosynthetic and pathogenic systems in *B. bassiana*.

## 2 Materials and methods

### 2.1 Fungal strain, insect, and culture conditions

*B. bassiana* BCC 2660 was obtained from the Thailand Bioresource Research Center in Thailand. The wild type and transformants were maintained on potato dextrose agar (PDA; Difco, United States) at 28°C. For *in vitro* experiments, *B. bassiana* wild type (WT *in vitro*) was cultured on Sabouraud Dextrose Broth (Difco, United States) supplemented with 1% yeast extract (SDY) at 150 rpm and 28°C for 3 days. For *in vivo* experiments, fungal conidia were harvested in saline (0.85% NaCl) and adjusted to 1 × 10^7^ cells mL^−1^. Fourth-instar beet armyworm (*Spodoptera exigua*) larvae were injected with 3 μL conidial suspension of *B. bassiana* wild type (WT *in vivo*) and *pks15-*null mutant (Δ*pks15 in vivo*), separately using a specialized 33-gauge needle-syringe set (Hamilton, United States). Fifty larvae and three replications for each treatment were conducted. Injected larvae were transferred individually into a 24-well plate and fed with armyworm medium, as described in a previous study (Abdullah et al., 2000).

### 2.2 RNA preparation and construction of cDNA library

Total RNA was extracted from WT *in vitro* (at 3 days post-inoculation), and WT *in vivo* and Δ*pks15 in vivo* at 48, 72 and 96 h post-infection using TRIzol Reagent (Sigma-Aldrich) and treated with DNase I (Thermo Scientific, United States). The mRNA was then purified from total RNA with Dynabeads^®^ mRNA Purification Kit (Thermo Scientific, United States), and mRNA integrity was assessed using Fragment Analyzer (Agilent^™^, United States). Purified mRNA was fragmented, and cDNA libraries were constructed using MGIEasy RNA Library Prep set (MGI Tech Co., Ltd., China) before sequencing with the MGISEQ-2000RS machine (MGI Tech).

### 2.3 *De novo* assembly and functional analysis of DEGs

Since the genome sequence of *B. bassiana* BCC 2660 has not been fully annotated, the transcriptomic read data were mapped to the reference genome of *B. bassiana* ARSEF 2860. The transcriptomic reads were aligned to the reference genome using Hisat2 version 2.2.0. Gene-level counts were generated by HT-seq count version 0.13.5. Differentially expressed genes (DEGs) of pairwise comparison between WT *in vivo* and WT *in vitro*, and Δ*pks15 in vivo* and WT *in vivo* were analyzed using DESeq2. Genes with a Benjamini–Hochberg-adjusted *p* < 0.01 were considered differentially expressed and categorised as up- or downregulated according to their log₂ fold change.

Gene Ontology (GO) categories and pathways were then assigned for functional categorization using ID mapping based on protein names in the UniProt Knowledgebase (UniProtKB)^1^ (Wang et al., 2021) with the cut-off threshold of 1 × 10^−3^.

### 2.4 Identification and generated protein networking of secondary metabolite biosynthetic gene clusters in *Beauveria bassiana* BCC2660

The whole genome of *B. bassiana* BCC 2660 with the accession number MWYT00000000 was entered into the antiSMASH fungal version^2^ to identify BGCs of secondary metabolites in this *B. bassiana* strain. The gene expression in BGCs of secondary metabolites in WT *in vivo* was compared to that of WT *in vitro* and to Δ*pks15 in vivo* at 48, 72, and 96 h of infection stages.

To determine the functional association in BGCs of secondary metabolites, the protein names in secondary metabolite BGCs were submitted to STRING version 12^3^ to generate protein networking with a full STRING network, maximum number of interactors set at 10, and high confidence (0.7). The differentially expressed genes (DEGs) were determined by pairwise comparison between WT *in vivo* vs. WT *in vitro*, and ∆*pks15 in vivo* vs. WT *in vivo* in the network.

## 3 Results

### 3.1 Differentially expressed genes in beet armyworms injected with *Beauveria bassiana* wild type (WT *in vivo*) and the ∆*pks15* mutant (∆*pks15 in vivo*)

**Figure 1:**
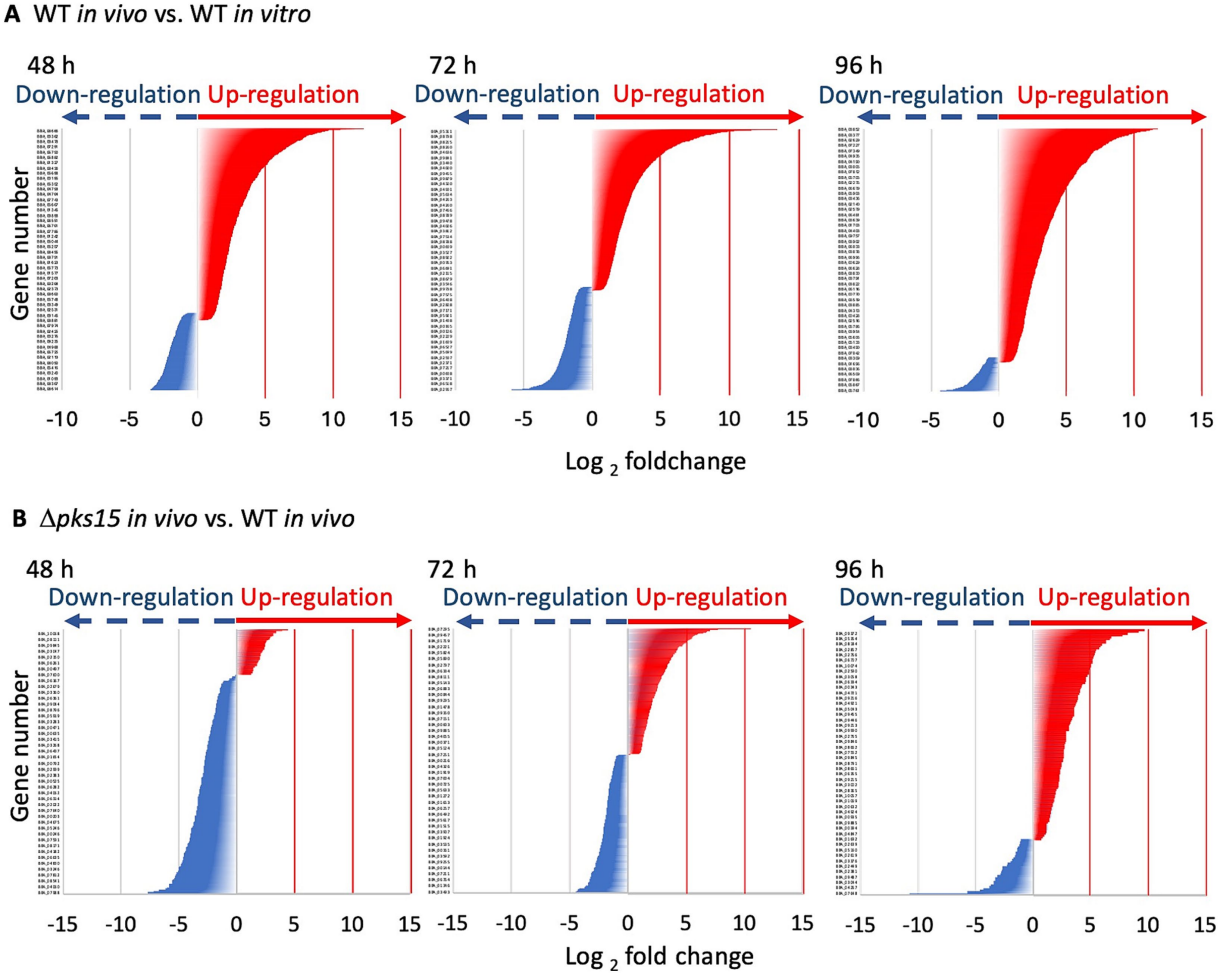
The numbers of up- and downregulated genes (red and blue, respectively) in the comparative analysis of WT *in vivo* vs. WT *in vitro* **(A)** and Δ*pks15 in vivo* vs. WT *in vivo* **(B)** across different infection stages (48, 72, and 96 h), along with their corresponding fold changes (*p* < 0.01). A large number of upregulated genes were observed in WT *in vivo* vs. WT *in vitro* throughout the infection period; whereas a substantial number of downregulated genes were detected in Δ*pks15 in vivo* vs. WT *in vivo*, particularly at 48 h during the early stage of infection.

To investigate the role of *pks15* in fungal development and the regulation of virulence factors during fungus-insect interactions, *in vivo* transcriptome analyses were conducted on *B. bassiana* wild-type injected-beet armyworm (WT *in vivo*) and Δ*pks15* mutant injected-beet armyworm (Δ*pks15 in vivo*). Infected larvae were collected at 3 time points: 48 h (fungal colonization of living larvae), 72 h (infected larvae deceased), and 96 h (fungal hyphae emerging from cadavers). Furthermore, an *in vitro* transcriptome analysis was conducted on *B. bassiana* wild type cultured in SDY (WT *in vitro*). For gene mapping, *B. bassiana* ARSEF 2860 was used as a reference. DEGs were identified by comparing the gene expression profiles of WT *in vivo* and WT *in vitro* (WT *in vivo* vs. WT *in vitro*) and Δ*pks15 in vivo* and WT *in vivo* (Δ*pks15 in vivo* vs. WT *in vivo*). In the WT *in vivo* and WT *in vitro* comparison at 48 h, 1,745 genes were differentially expressed, with 1,192 upregulated genes and 553 downregulated genes. The DEG number increased to 1,622 genes at 72 h, with 1,522 upregulated genes and 100 downregulated genes. This number decreased to 1,082 genes at 96 h, with 943 upregulated genes and 139 downregulated genes. In contrast, lower numbers of DEGs were identified in the Δ*pks15 in vivo* and WT *in vivo* comparison. Numbers of DEG were 225, 399 and 207 genes at 48, 72 and 96 h, respectively. At 48 h, 39 genes were upregulated, and 186 genes were downregulated. At 72 h, 190 genes were upregulated, and 209 genes were downregulated. Finally, at 96 h, the numbers of up- and downregulated genes decreased to 116 and 91, respectively (Figure 1).

### 3.2 Functional annotation of DEGs in WT *in vivo* (WT *in vivo* vs. WT *in vitro*) and Δ*pks15 in vivo* (Δ*pks15 in vivo* vs. WT *in vivo*)

In Gene Ontology (GO) analysis of DEGs, the number of genes annotated in three GO categories, “biological process,” “cellular component,” and “molecular function,” was assessed. DEGs in WT *in vivo* (WT *in vivo* vs. WT *in vitro*) were categorized to have proportions of 37, 37, and 31% for biological process, 49, 43 and 34% for cellular component, and 59, 58, and 56% for molecular function at 48, 72, and 96 h, respectively. DEGs in Δ*pks15 in vivo* (Δ*pks15 in vivo* vs. WT *in vivo*) were annotated with respective proportions of 34, 37, and 29% for biological process, 26, 40, and 29% for cellular component, and 52, 54, and 61% for molecular function, at 48, 72, and 96 h, respectively. Comparative analysis revealed distinct categories of genes upregulated in WT *in vivo* and downregulated in Δ*pks15 in vivo* during various infection stages within the top GO categories related to biological processes, cellular components, and molecular function.

**Figure 2:**
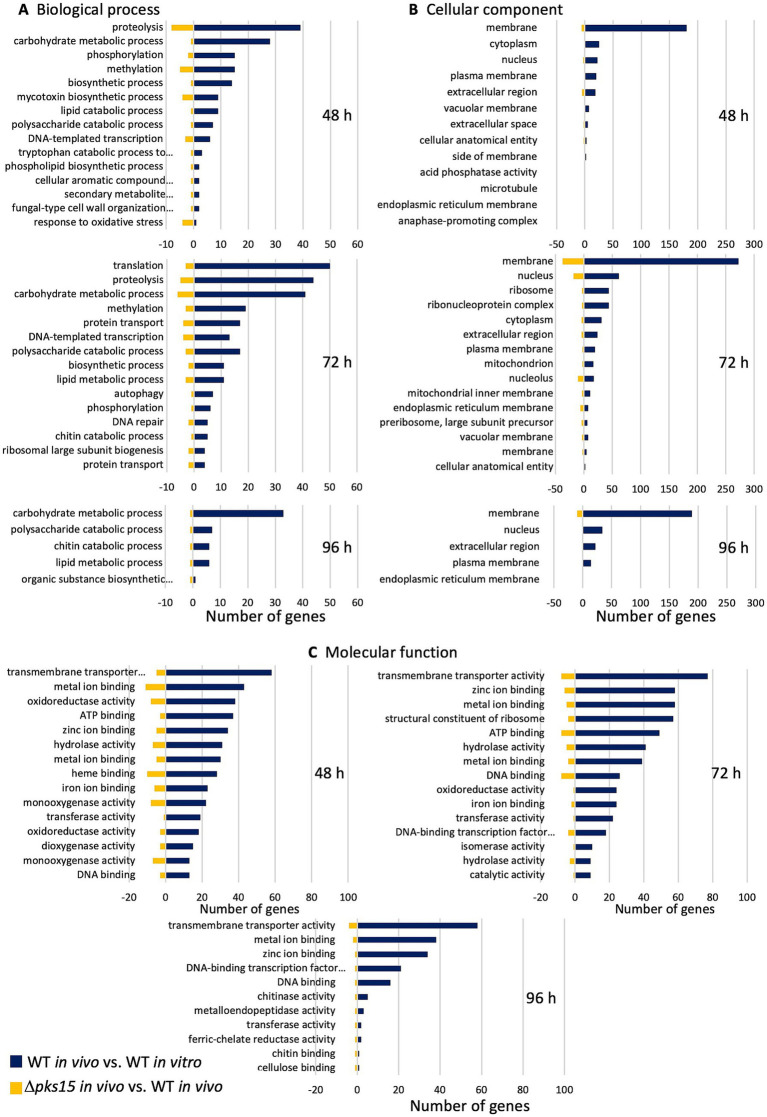
The top Gene Ontology (GO) categories, **(A)** biological process, **(B)** cellular component, and **(C)** molecular function, represented as the numbers of upregulated (blue) and downregulated (yellow) genes in the comparative analysis of WT *in vivo* vs. WT *in vitro* and Δ*pks15 in vivo* vs. WT *in vivo*, respectively, at 48, 72, and 96 h.

The top GO categories with large numbers of genes within the biological process category for upregulated genes in WT *in vivo* (WT *in vivo* vs. WT *in vitro*) and downregulated genes in Δ*pks15 in vivo* (Δ*pks15 in vivo* vs. WT *in vivo*) were identified. These categories included proteolysis (GO:0006508), carbohydrate metabolic process (GO:0005975), methylation (GO:0032259), biosynthetic process (GO:0009058), lipid metabolic process (GO:0006629), polysaccharide catabolic process (GO:0000272), and DNA-templated transcription (GO:0006351) at both 48 and 72 h. Similarly, at 96 h, the carbohydrate metabolic process, polysaccharide catabolic process, and lipid metabolic process remained prominent. In addition, chitin catabolic process (GO:0006032) and organic substance biosynthetic process (GO:1901576) were observed at this time point. Genes were involved in virulence, secondary metabolite production, stress response and insect immune response were identified within GO terms related to phosphorylation (GO:0016310), mycotoxin biosynthetic process (GO:0043386), tryptophan catabolic process to kynurenine (GO:0019441), cellular aromatic compound metabolic process (GO:0006725), secondary metabolite biosynthetic process (GO:0044550), and fungal-type cell wall organization or biogenesis (GO:0071852) at 48 h. Additional processes, including protein transport (GO:0015031) and autophagy (GO:0006914), were annotated at 72 h (Figure 2A). Various genes involved in these GO categories encoded protein kinase domain-containing protein, twin-arginine translocation (Tat) pathway signal sequence and oxidase *ustYa*, indoleamine 2,3-dioxygenase, vacuolar fusion protein MON1, vacuolar protein sorting-associated protein VTA1, ESCRT-II complex subunit, protein SDA1 and SNARE domain-containing protein.

The largest number of genes associated with GO cellular component categories, including genes upregulated in WT *in vivo* (WT *in vivo* vs. WT *in vitro*) and down-regulated in Δ*pks15 in vivo* (Δ*pks15 in vivo* vs. WT *in vivo*), were mapped to membrane (GO:0016020), plasma membrane (GO:0005886), extracellular region (GO:0005576), and nucleus (GO:0005634) throughout the infection period. Notably, genes involved in pathogenicity and associated with the production of secondary metabolites were annotated under nucleus (GO:0005634), cellular anatomical entity (GO:0110165), extracellular region (GO:0005576), extracellular space (GO:0005615), membrane (GO:0016020), and plasma membrane (GO:0005886) (Figure 2B). These genes encoded transcription factors (C6 transcription factors, fungal-specific transcription factors, and BZIP transcription factors), transporters [major facilitator superfamily (MFS) transporters, siderophore iron transporter, and ATP-binding cassette (ABC) transporters] and cytochrome P450.

The top GO categories of molecular functions with genes upregulated in WT *in vivo* (WT *in vivo* vs. WT *in vitro*) and down-regulated in Δ*pks15 in vivo* (Δ*pks15 in vivo* vs. WT *in vivo*) were related to transmembrane transporter activity (GO:0022857), zinc ion binding (GO:0008270), metal ion binding (GO:0046872), transferase activity (GO:0016740), DNA-binding transcription factor activity, RNA polymerase II-specific (GO:0000981), DNA binding (GO:0003677), and cellulose binding (GO:0030248) were annotated throughout the infection period. In contrast, oxidoreductase activity (GO:0016491), iron ion binding (GO:0005506), ATP binding (GO:0005524), hydrolase activity (GO:0016787), and catalytic activity (GO:0003824) were specifically observed at 48 h and 72 h. Genes for chitinase activity (GO:0004568) and metalloendopeptidase activity (GO:0004222) were also found at 72 and 96 h (Figure 2C). Genes involved in virulence and biosynthesis and export of secondary metabolites were annotated under terms such as metal ion binding (GO:0046872), methyltransferase activity (GO:0008168), oxidoreductase activity (GO:0016491), S-adenosylmethionine-dependent methyltransferase activity (GO:0008757) and transmembrane transporter activity (GO:0022857). These genes encoded RadH flavin-dependent halogenase, flavin-binding monooxygenase, efflux pump antibiotic resistance protein, glutathione S- transferase OpS6, 1,3-beta-glucanosyltransferase, MFS transporters, siderophore iron transporter, and amino acid permeases.

### 3.3 A large number of genes involved in the mycotoxin biosynthesis, secondary metabolite biosynthesis, and glycan metabolism pathways were upregulated in WT *in vivo* (WT *in vivo* vs. WT *in vitro*) and downregulated in Δ*pks15 in vivo* (Δ*pks15 in vivo* vs. WT *in vivo*)

**Figure 3:**
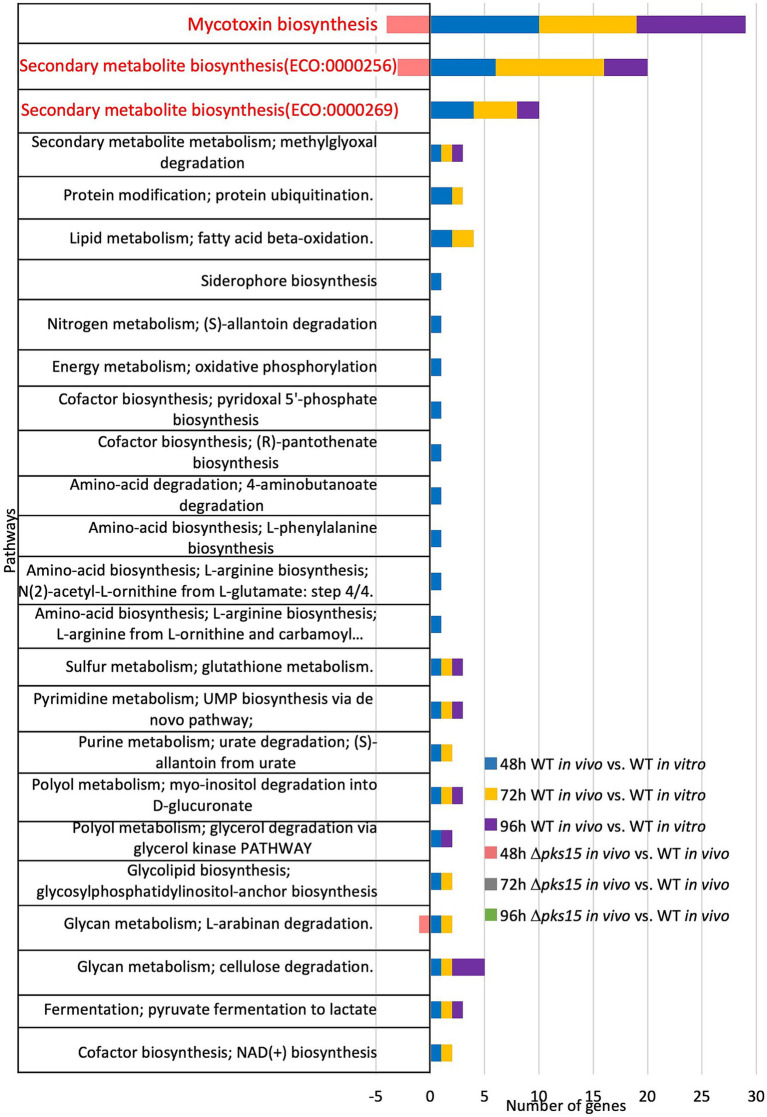
The top three pathways, one mycotoxin biosynthesis and two secondary metabolite biosynthetic pathways, represented as the numbers of upregulated and downregulated genes in the comparative analysis of WT *in vivo* vs. WT *in vitro* and Δ*pks15 in vivo* vs. WT *in vivo*, respectively, at 48, 72, and 96 h.

The top three pathways identified in WT *in vivo* during the infection of beet armyworm were one pathway related to mycotoxin biosynthesis (ECO:0000256) and two pathways associated with secondary metabolite biosynthetic pathways (ECO:0000256 and ECO:0000269). In mycotoxin biosynthesis, 10, 9, and 10 mycotoxin-related genes were upregulated in WT *in vivo* compared to WT *in vitro* at 48, 72, and 96 h, respectively. Notably, four of these 10 genes were downregulated in Δ*pks15 in vivo* compared to WT *in vivo* at 48 h. For secondary metabolite biosynthesis, 10, 14, and 6 genes were upregulated in WT *in vivo* relative to WT *in vitro*, at 48, 72, and 96 h, respectively, while three genes were down-regulated in Δ*pks15 in vivo* relative to WT *in vivo* at 48 h. Additionally, alpha-L-arabinofuranosidase gene (BBA_06314), which is involved in the glycan metabolism pathway, specifically L-arabinan degradation, was upregulated by 1.8-fold in WT *in vivo* (versus WT *in vitro*) at 48 and 72 h. In contrast, this gene was downregulated 3.3-fold in Δ*pks15 in vivo* (versus WT *in vivo*) at 48 h (Figure 3).

### 3.4 DEGs in mycotoxin biosynthesis, secondary metabolite biosynthesis, and cell wall synthesis pathways in *Beauveria bassiana* WT

**Figure 4:**
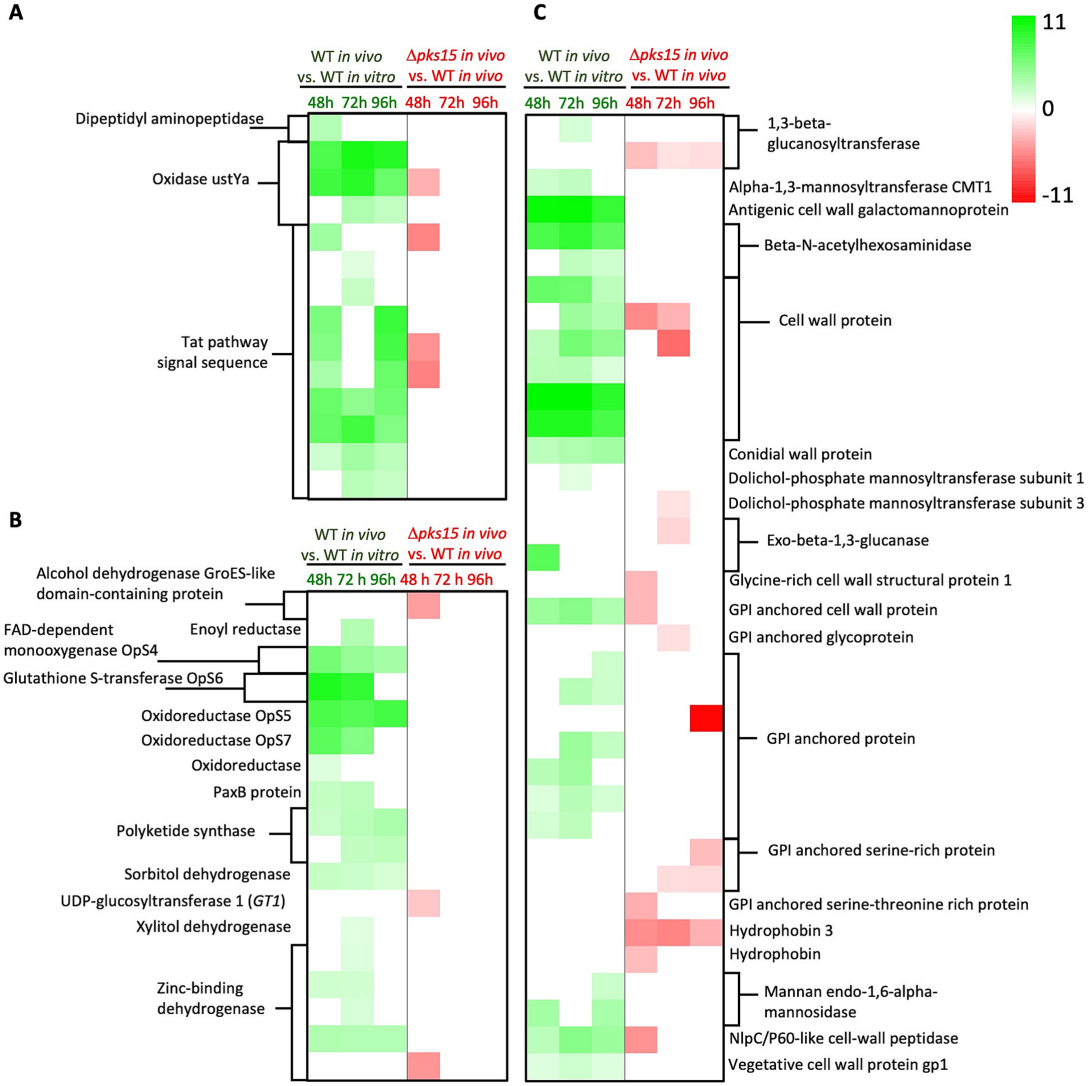
A heatmap showing log_2_ fold changes of DEGs upregulated (green gradient) and downregulated (red gradient) in the comparative analysis of WT *in vivo* vs. WT *in vitro* and Δ*pks15 in vivo* vs. WT *in vivo*. Genes related to mycotoxin biosynthesis **(A)**, secondary metabolite biosynthesis **(B)**, and cell wall synthesis **(C)** at 48, 72, and 96 h (*p* < 0.01) are highlighted.

DEGs in the three predominant pathways were explored more specifically in WT *in vivo* and Δ*pks15 in vivo* at different host infection stages. For mycotoxin biosynthesis, three genes encoding oxidase *ustYa* (BBA_04660, BBA_07639, and BBA_09879) were upregulated with fold increases of 8, 3–10 and 3–9 at 48, 72, and 96 h, respectively, in WT *in vivo* relative to WT *in vitro*. In contrast, one gene of the oxidase *ustYa* (BBA_07639) was downregulated 3–fold at 48 h in Δ*pks15 in vivo* relative to WT *in vivo*. In addition, 10 genes annotated to Tat pathway signal sequence (BBA_00196, BBA_02514, BBA_03661, BBA_04184, BBA_04188, BBA_04190, BBA_07364, BBA_07638, BBA_09877, and BBA_09880) were upregulated by 2–7, 1–8 and 2–9 fold at 48, 72, and 96 h, respectively, in WT *in vivo* compared to WT *in vitro*, while three Tat-pathway signal sequence genes (BBA_00196, BBA_04188, and BBA_04190) in Δ*pks15 in vivo* compared to WT *in vivo* were down-regulated 5-fold. The dipeptidyl aminopeptidase gene (BBA_09990) was upregulated 3-fold in WT *in vivo* relative to WT *in vitro* (Figure 4A and Supplementary Table S1).

For secondary metabolite biosynthesis, several genes in oosporein biosynthesis were upregulated during infection and colonization of insects 6–10, 5–9 and 2–8-fold in WT *in vivo* (vs. WT *in vitro*) at 48, 72, and 96 h, respectively. These genes included oosporein biosynthesis protein 4 *OpS4* (FAD-dependent monooxygenase), *OpS5* (laccase), *OpS6* (glutathione S-transferase), and *OpS7* (oxidoreductase). In addition, several other genes were notable, including the polyketide synthase *pks3* (BBA_09745) upregulated 2, 3, and 4-fold, four genes encoding zinc-binding dehydrogenases (BBA_00728, BBA_01623, BBA_04759, and BBA_06349) upregulated 2–3, 1–3, and 3-fold, and a sorbitol dehydrogenase gene (BBA_01242) upregulated 2-fold in WT *in vivo* at 48, 72, and 96 h, respectively. An oxidoreductase gene (BBA_09885) was upregulated 1.5-fold at 48 h, while paxillin B gene *PaxB* (BBA_09378) showed a 3-fold increase at both 48 and 72 h. Genes for an enoyl reductase (BBA_00966) and a xylitol dehydrogenase (BBA_04382) were upregulated 3 and 1.3-fold, respectively, at 72 h. Another polyketide synthase, *pks5* (BBA_08219) was increased 3-fold at both 72 and 96 h. In contrast, several genes were downregulated in Δ*pks15 in vivo* (vs. WT *in vivo*) at 48 h. These included an alcohol dehydrogenase gene (GroES-like domain-containing protein, BBA_06625), with a 4-fold decrease, UDP-glucosyltransferase 1 gene, *GT1* (BBA_08686) with a 2-fold decrease, and a zinc-binding dehydrogenase gene (BBA_08790) that exhibited a 4.5-fold decrease (Figure 4B and Supplementary Table S2).

In addition, numerous genes associated with cell wall synthesis and structure were differentially expressed, with 25 genes upregulated in WT *in vivo* (vs. WT *in vitro*) and 15 genes downregulated in Δ*pks15 in vivo* (vs. WT *in vivo*). Six genes encoding cell wall proteins (BBA_02602, BBA_03246, BBA_03493, BBA_05808, BBA_08793, and BBA_08794) exhibited 3- to 11-, 3- to 11-, and 2- to 9-fold increases at 48 h, 72 h, and 96 h, respectively, in WT *in vivo* compared to WT *in vitro*. In contrast, two cell wall protein genes (BBA_03246 and BBA_03493) were downregulated by 5- and 3- to 6-fold at 48 h and 72 h, respectively, in Δ*pks15 in vivo* compared to WT *in vivo*. This downregulation of BBA_03246 and BBA_03493 in Δ*pks15* vs. WT *in vivo* was concomitant with the upregulation of these two genes in WT *in vivo* vs. *in vitro*. The 1,3-beta-glucanosyltransferase gene (BBA_03082) showed a 2-fold increase in expression at 72 h in WT *in vivo*, but BBA_05160 decreased 3, 1.2, and 1.4-fold at 48, 72 and 96 h, respectively, in Δ*pks15 in vivo*. Furthermore, expression of dolichol-phosphate mannosyltransferase subunit 1 gene (BBA_08658) increased 1.3-fold at 72 h in WT-BAW, whereas that of the subunit 3 gene (BBA_05676) was downregulated 1.2-fold at 48 h in Δ*pks15 in vivo*. Exo-beta-1,3-glucanase gene (BBA_09211) expression increased 7-fold at 48 h in WT *in vivo*, but decreased 2-fold at 72 h in Δ*pks15 in vivo* (BBA_02045). Glycosylphosphatidylinositol (GPI)-anchored cell wall protein gene (BBA_00525) was upregulated 4, 5, and 3-fold at 48, 72, and 96 h, respectively, in WT *in vivo*, but downregulated 3-fold at 48 h in Δ*pks15 in vivo*. NlpC/P60-like cell-wall peptidase gene (BBA_07693) was upregulated 3, 5, and 4-fold at 48, 72 and 96 h, respectively, in WT *in vivo*, but downregulated 5-fold at 48 h in Δ*pks15 in vivo* (Figure 4C and Supplementary Table S3).

Additionally, expression of seven cell wall-associated genes increased in WT *in vivo*, compared with WT *in vitro*. At 48, 72, and 96 h, cell wall galactomannoprotein gene (BBA_02996) was markedly upregulated 11, 11, and 9-fold, respectively, while two genes encoding beta-N-acetylhexosaminidases (BBA_02233 and BBA_07539) were upregulated 8, 3–9, and 2-7-fold. Conidial wall protein gene (BBA_07138) was upregulated 3, 3.5, and 4-fold and vegetative cell wall protein gene *gp1* (BBA_09190) was upregulated 1.5, 2, and 1.4-fold. Increased expression of mannan endo-1,6-alpha-mannosidase gene (BBA_06352) by 4 and 3.7-fold was detected at 48 h and 96 h, respectively, while alpha-1,3-mannosyltransferase CMT1 gene (BBA_01149) was upregulated 2.2 and 2.6-fold over the same time periods (Figure 4C and Supplementary Table S3). Intriguingly, these DEGs were not found in Δ*pks15 in vivo* vs. WT *in vivo*.

In contrast, seven genes were downregulated in Δ*pks15 in vivo* (vs. WT *in vivo*), whereas no DEGs were detected in WT *in vivo* (vs. WT *in vitro*). The downregulated genes included glycine-rich cell wall structural protein 1 gene (BBA_06376), which was decreased 3-fold at 48 h, and GPI-anchored glycoprotein gene (BBA_01335), which was decreased 1.4-fold at 72 h. Two genes encoding GPI-anchored serine-rich proteins (BBA_07048 and BBA_00643) were decreased 1.5- and 1.5- to 3-fold at 72 h and 96 h, respectively. GPI-anchored serine-threonine-rich protein-encoding gene (BBA_02631) was downregulated 3-fold at 48 h. Hydrophobin 3 gene (BBA_07597) was downregulated 5-, 5-, and 3-fold at 48 h, 72 h, and 96 h, respectively, while hydrophobin-like protein-encoding gene (BBA_02999) was downregulated 3-fold at 48 h (Figure 4C and Supplementary Table S3).

### 3.5 Various genes in secondary metabolite biosynthesis clusters were upregulated in WT *in vivo* (WT *in vivo* vs. WT *in vitro*) and downregulated Δ*pks15 in vivo* (Δ*pks15 in vivo* vs. WT *in vivo*)

**Figure 5:**
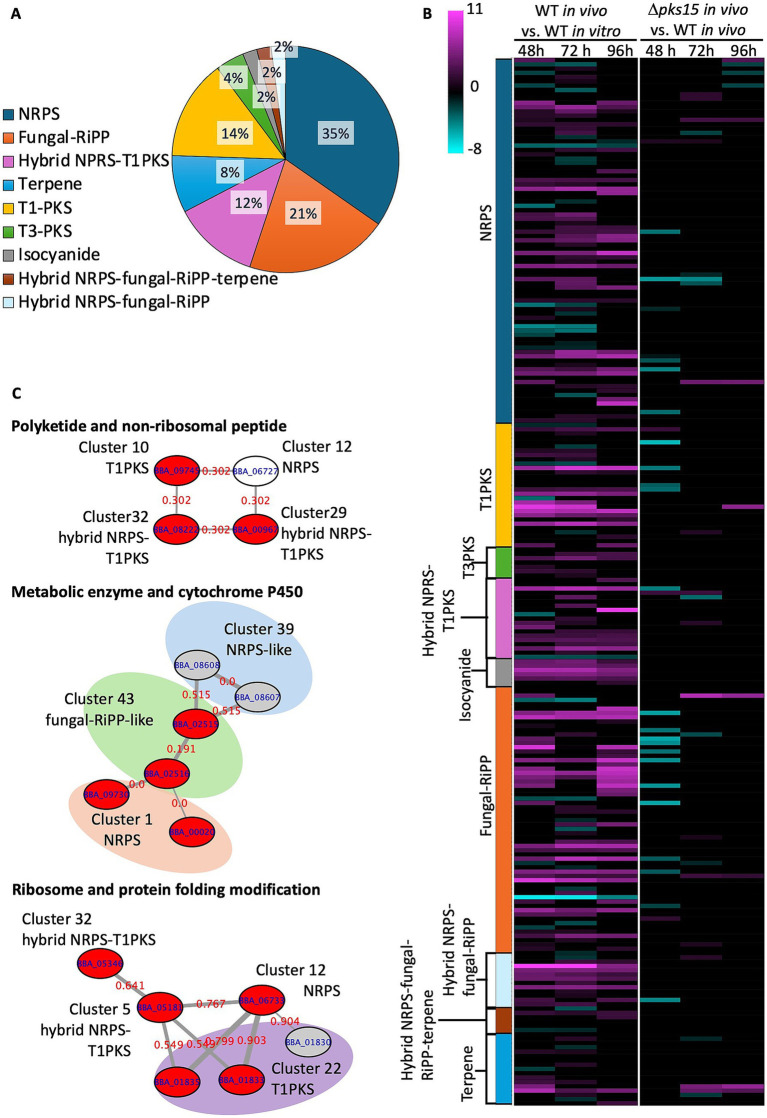
Secondary metabolite biosynthetic clusters annotated by antiSMASH in *B. bassiana* BCC 2660. **(A)** Clusters of different types of secondary metabolites and their proportions. **(B)** Heatmap of expressed genes from different types of secondary metabolite BGCs showing upregulated genes (pink gradient) and downregulated genes (green gradient) in the comparative analysis of WT *in vivo* vs. WT *in vitro* and Δ*pks15 in vivo* vs. WT *in vivo* at 48, 72, and 96 h (*p* < 0.01). **(C)** Protein–protein interactions analyzed by STRING represent coordinated up (red nodes) and down (grey nodes) regulation of genes in WT *in vivo*. White nodes represent genes that had no differential expression.

To reveal the expression levels of genes involved in secondary metabolite biosynthesis, the whole genome of *B. bassiana* BCC 2660 (accession number MWYT00000000) was analyzed, revealing 45 clusters through the antiSMASH fungal version (accessed on 01 July 2024). All the clusters comprised 575 genes, which were classified into 17 nonribosomal peptide synthetases (NRPSs) (35%), 10 fungal-RiPP clusters (20%), 6 hybrid NRPS- type I-polyketide synthases (T1-PKS) clusters (12%), 4 terpene clusters (8%), 7 T1-PKSs (14%), 2 type III PKSs (T3-PKSs; 4%), 1 hybrid NRPS-fungal RiPP-terpene cluster (2%), 1 hybrid NRPS-fungal RiPP cluster (2%), and 1 isocyanide (ICS) cluster (2%). One hundred and eighty-seven out of 575 genes in 45 clusters exhibited differential expression (Figure 5A). The heatmap analysis (Figure 5B) showed 151 genes in the secondary metabolite clusters, which were upregulated in WT *in vivo* across different time points (pink colour). These genes were defined into various functional groups, including cytochrome P450 family, transcription factors, membrane proteins, secondary metabolite biosynthesis, oxidoreductases and dehydrogenases, proteolysis, signal peptides, ubiquitin-proteasome system, lipid binding/transport, chaperones and protein folding, transporters, enzymes and catalytic proteins, structural cell wall and other functionally diverse proteins, cell division and regulatory proteins and hypothetical/ uncharacterized proteins. In comparison to WT *in vivo*, Δ*pks15 in vivo* exhibited 24, 9, and 3 downregulated genes at the early stage (48 h), middle stage (72 h) and late stage (96 h) of infection, respectively (green colour, Supplementary Table S4). These downregulated genes were annotated in the functional categories of signal peptides, catalytic enzymes, secondary metabolite biosynthesis, transporters, structural cell wall proteins, cell division and regulatory proteins, chaperones and protein folding, and uncharacterized/hypothetical proteins. These DEGs are all shown in Supplementary Table S4.

Among the secondary metabolite BGCs in *B. bassiana* BCC 2660, we focused on elucidating the expression patterns of the genes in the *pks15* cluster (T1PKS, cluster 30) and *pks14* cluster (T1PKS-NRPS, cluster 29), as these two PKS genes were previously demonstrated to be important for insect pathogenicity (Toopaang et al., 2017; Srisuksam et al., 2018). Fascinatingly, transcriptome analysis indicated that the UDP-glucosyl transferase *Bbugt1* in *pks15* cluster was upregulated in WT *in vivo* relative to WT *in vitro* at the three time points, but downregulated in the *∆pks15 in vivo* relative to WT *in vivo* at the early stage of infection (48 h). In the *pks14* cluster, the main PKS gene “*pks14*,” a monooxygenase gene, an enoyl reductase gene and a gene encoding a hypothetical protein (ORF10) were upregulated. This result is consistent with our previous finding that the gene *pks14* was expressed only in the insect-containing culture (Srisuksam et al., 2018).

Protein–protein interactions were observed across multiple clusters of secondary metabolites using STRING (accessed on 1 July 2024). Their cross-relationships were illustrated as networks connecting T1-PKS, hybrid NRPS-T1PKS, fungal-RiPP, and NRPS genes, with enhanced expression found in WT *in vivo* relative to WT *in vitro*. One heterogeneous network consisted of one T1-PKS gene (BBA_09745 in cluster 10), an NRPS gene (BBA_06727 in cluster 12), and two hybrid NRPS-T1-PKS genes (BBA_00967 and BBA_08222 from the clusters 29 and 32, respectively). All these genes showed increased expression from 72 h to the later stage of infection at 96 h. A second network highlighted an association between metabolic enzymes and cytochrome P450. This network included a citrate synthase family member gene (BBA_02515) and a malate synthase gene (BBA_02516) (both from cluster 43 of fungal RiPP), a methylmalonate-semialdehyde dehydrogenase gene (BBA_09730) and a cytochrome P450 CYP5293A2 gene (BBA_09720) (from cluster 1 involved in beauvericin biosynthesis). These genes exhibited increased expression during the early, middle and late stages of infection. A third network linked ribosome-related and protein folding modification genes, comprising a ureidoglycolate hydrolase gene (BBA_01835), a 60S ribosomal protein L44 gene (BBA_01833) (from cluster 22 of T1-PKS), a ribosomal S4 family gene (BBA_06733) (from cluster 12 of NRPS), an alpha/beta fold family gene (BBA_05181) (from cluster 5 of hybrid NRPS-T1PKS) and an FtsJ-like methyltransferase gene (BBA_05346) (from cluster 32 of hybrid NRPS-T1PKS). These genes displayed enhanced expression from the early to middle stages of infection (Figure 5C and Supplementary Table S4). Notably, these genes that were differentially expressed within the networks were absent in the comparative analysis of Δ*pks15* vs. WT, under *in vivo* conditions. These results demonstrated the functional associations between different BGCs involved in secondary metabolite production. The co-expression of genes within clustered networks of T1-PKS, NRPS, hybrid NRPS-T1PKS, and fungal-RiPP clusters suggested a coordinated upregulation of these genes during infection.

### 3.6 Cross-pathway impact of the *pks15* cluster on DEGs in other secondary metabolite BGCs

**Figure 6:**
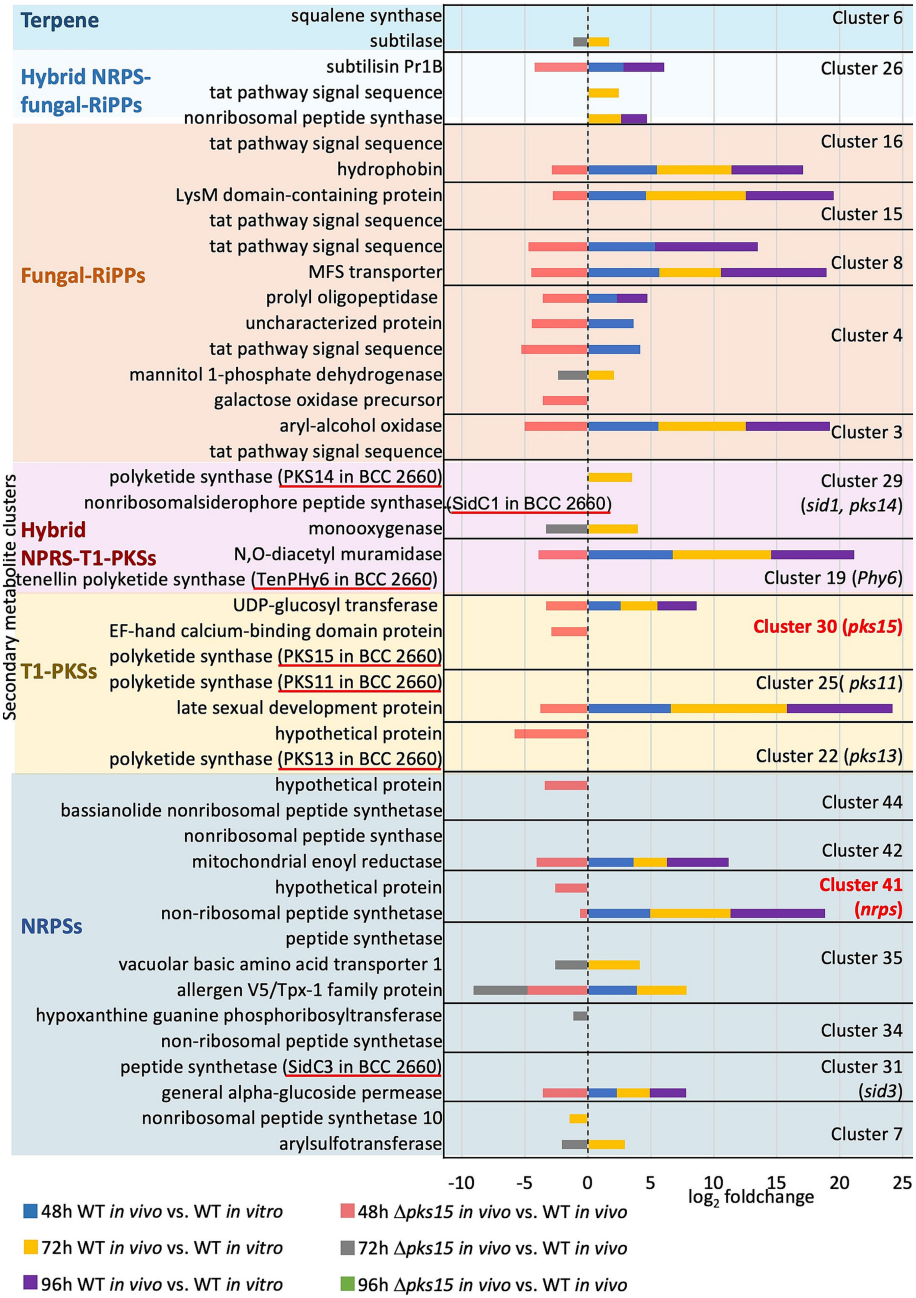
The *pks15* cluster influences the expression of genes in other secondary metabolite BGCs. DEGs upregulated in WT *in vivo* (compared to WT *in vitro*) and downregulated in Δ*pks15 in vivo* (compared to WT *in vivo*) at 48, 72, and 96 h (*p* < 0.01) are shown.

To investigate the influence of *pks15* expression on crosstalk between genes from different secondary metabolite BGCs, DEGs from secondary metabolite clusters were considered in the Δ*pks15 in vivo* strain, where the PKS gene had been deleted. Twenty-two genes from the “*pks15*” cluster 30, NRP clusters 7, 31, 35, 41, and 42, T1-PKS clusters 25, hybrid NRP-T1PKS clusters 19 and 29, fungal-RiPP clusters 3, 4, 8, 11, 15, and 16, hybrid NRP, fungal-RiPP cluster 26, and terpene cluster 6 were upregulated in the WT *in vivo* (vs. WT *in vitro*) 2–9 fold throughout the infection period, but noticeably downregulated in Δ*pks15 in vivo* (vs. WT *in vivo*) 1–5 fold at 48 h and 72 h (Figure 6 and Supplementary Table S5). These genes included a UDP-glucosyl transferase gene (BBA_01012) in the “*pks15*” cluster 30, a arylsulfotransferase gene (BBA_04027), an alpha-glucoside permease gene (BBA_08241), an allergen V5/Tpx-1 family gene (BBA_04830), a vacuolar basic amino acid transporter 1 gene (BBA_04828), a nonribosomal peptide synthetase gene (BBA_07611) and a mitochondrial enoyl reductase gene (BBA_07591) from the NRP clusters, as well as a late sexual development gene (BBA_03813) from another T1-PKS cluster. Other DEGs included an N,O-diacetyl muramidase gene (BBA_07331) and a monooxygenase gene (BBA_00962) from the hybrid NRP-T1PKS clusters. Other DEGs from the fungal-RiPP clusters included an aryl-alcohol oxidase gene (BBA_00090), a mannitol 1-phosphate dehydrogenase gene (BBA_00195), a Tat pathway signal sequence gene (BBA_00196 and BBA_00196), a gene encoding an uncharacterized protein (BBA_00197), a prolyl oligopeptidase family gene (BBA_00203), an MFS transporter gene (BBA_04182), a LysM domain-containing gene (BBA_02945), and a hydrophobin gene (BBA_02999). These genes also included a subtilisin gene *pr1B* (BBA_03653) from the hybrid NRP-fungal-RiPP cluster, and a subtilase gene (BBA_05303) from the terpene cluster. Intriguingly, the downregulation of a NRPS gene (BBA_07611) by 0.6-fold and a gene encoding a hypothetical protein (BBA_07610) by 2.3-fold from the cluster 41, and the downregulation of a gene of uncharacterized function (BBA_02631) by 3.4-fold, in the bassianolide cluster 44, underscored the critical roles of different secondary metabolites in the host infection.

Additionally, six genes were downregulated in Δ*pks15 in vivo* compared with WT *in vivo*. These genes were associated with the purine salvage pathway (BBA_05682), calcium ion binding (BBA_01011), galactose oxidation (BBA_00193), and encoding two hypothetical proteins (BBA_02631 and BBA_01838). These DEGs play a critical role in nonribosomal peptide and polyketide biosynthesis, transport and permease activity, glycosylation and sulfation, oxidation and dehydrogenation, proteolysis, peptidase activity, signal peptides, and cell wall and surface proteins (Figure 6 and Supplementary Table S5). Some DEGs encoded uncharacterized proteins with no known functions. Our data emphasized the impact of PKS15 on the coordinated regulation of other secondary metabolite BGCs during the infection. Together, these genes play roles in pathogenicity, immune evasion, and detoxification, which contribute to the pathogenicity of *B. bassiana* in insect hosts.

Overall, this comparative transcriptomic analysis revealed that PKS15 or its associated polyketide product plays an essential role in regulating virulence-associated pathways critical for fungal infection. Genes involved in mycotoxin production and secondary metabolite biosynthesis were markedly upregulated in WT *in vivo* but suppressed in the ∆*pks15* mutant, highlighting PKS15’s broad regulatory influence across BGCs beyond its own cluster. Likewise, genes essential for fungal cell wall remodeling and immune evasion were downregulated in the ∆*pks15* mutant, emphasizing PKS15’s contribution to metabolic adaptation and pathogenicity. Importantly, transcriptomic profiling uncovered extensive co-regulation and functional crosstalk between the *pks15* cluster and diverse secondary metabolite BGCs, including NRPS, hybrid NRPS-T1PKS, fungal RiPP, and terpene clusters.

## 4 Discussion

During insect pathogenesis in the hemocoel, fungal pathogens utilize various mechanisms to initiate infection, overcome host defenses, and ultimately cause insect death. This study elucidated some key genes from the entomopathogenic fungus *Beauveria bassiana* through comparative transcriptomic analysis. Two primary comparisons were performed: *B. bassiana* wild-type *in vivo* versus wild-type *in vitro*, and *B. bassiana* wild-type *in vivo* versus Δ*pks15 in vivo*. The results revealed that secondary metabolite biosynthesis and cell wall-associated genes are among the most prominently upregulated groups in the WT *in vivo* condition compared to both *in vitro* culture and the Δ*pks15 in vivo*, in which the polyketide synthase gene *pks15* is deficient. These findings furthermore suggest that infection within the insect hemocoel involves the production of several secondary metabolites and the construction or reconstruction of the fungal cell wall. Our data are consistent with a previous report that *B. bassiana* PKS15 exhibited the crosstalk with other secondary metabolite BGCs, including those for biosynthesis of beauvericin, bassianolide, enniatin, and intracellular siderophore (Toopaang et al., 2023).

Among the genes associated with secondary metabolite and mycotoxin biosynthesis, the oxidase gene *ustYa*, a Tat pathway signal sequence-containing gene, a flavin-binding monooxygenase gene, a glutathione S-transferase gene and siderophore transporter genes were upregulated in WT *in vivo* relative to WT *in vitro* and downregulated in Δ*pks15 in vivo* relative to WT *in vivo*, particularly during the early stages of infection. At this stage (48 h post-inoculation), the insect immune responses, such as hemocyte accumulation, are triggered against fungal invasion. In response, the fungus overcomes these insect defenses, colonizes the host, and produces high levels of bioinsecticides and virulence factors such as beauvericin, bassianolide, and ferricrocin. Therefore, the upregulation of secondary metabolite and mycotoxin biosynthetic genes is essential for overcoming the insect immune response and successful colonization (Bidochka and Khachatourians, 1987; Travis et al., 1995; Toopaang et al., 2017; Udompaisarn et al., 2020; Toopaang et al., 2023).

UstYa is an oxidase commonly found in fungal pathogens. It is involved in the biosynthesis of ustiloxins, which are cyclic peptidyl secondary metabolites, and mycotoxins such as ustiloxin B and ustiloxin F in *A. flavus* (Nagano et al., 2016). Ustiloxins are produced in the early stages of plant infection and may contribute to pathogenicity (Hu et al., 2020). The Tat pathway is a transport system that plays a critical role in the virulence factor of bacterial pathogens, although its function in fungal pathogens remains unclear. The Tat system is involved in colonization, iron acquisition, and contributes to tumour formation of plant pathogens such as *Yersinia pseudotuberculosis* (De Buck et al., 2005; Lavander et al., 2006), *Pseudomonas aeruginosa*, *Legionella pneumophila* (De Buck et al., 2008) and *Agrobacterium tumefaciens* (Ding and Christie, 2003). Deletion of the Tat system results in deficiency in siderophore production, decreased stress resistance, and reduced fitness of the *P. syringae* in plant hosts (Caldelari et al., 2006). In the plant pathogenic fungus *Alternaria brassicicola*, the flavin-binding monooxygenase AbMak1 is involved in cell wall biogenesis and influences the melanization process (Pigné et al., 2017). Glutathione transferases (GSTs) play an essential role in protecting plant pathogenic fungi from plant-produced toxic metabolites through detoxification and tolerance to oxidative stress (Calmes et al., 2015). Siderophores are well-known virulence factors in the entomopathogenic fungus *B. bassiana*, enabling the acquisition of iron from iron-deficient environments, thus promoting the pathogenicity of fungal pathogens (Khasheii et al., 2021). In the human pathogenic fungus *C. albicans*, siderophore transporters, Sit1p/Arn1p are not only responsible for the uptake of the ferrichrome-type siderophores, but are also involved in the epithelial invasion and penetration of mucosal model (Heymann et al., 2002).

A previous study demonstrated that PKS15 is associated with the structural integrity of the cell wall. The Δ*pks15* mutant lost the rodlet structure on the cell surface, which compromised its anti-phagocytic ability and ability to escape from insect hemocytes (Toopaang et al., 2017). In this study, transcriptome analysis confirmed that some genes associated with cell wall structure were downregulated in Δ*pks15*-BAW. These genes included a 1,3-beta-glucanosyltransferase gene, an exo-beta-1,3-glucanase gene, a GPI-anchored cell wall gene, and a hydrophobin gene, which are essential for cell wall integrity, host infection, stress response, and evasion of host immune defenses. The l,3-β-glucanosyltransferases, commonly found in yeast and fungi, contribute to the elongation of l,3-β-glucan chains (Hartland et al., 1996) and play a crucial role in maintaining cell wall integrity in both spores and hyphae. The deletion of this gene reduces fungal growth and cell aggregation, alters cell wall composition and decreases infectivity in fungal pathogens (Popolo et al., 1993; Popolo and Vai, 1999; Mouyna et al., 2005). GPI-anchored proteins are involved in signaling, cell adhesion, cell wall metabolism and integrity, and immune response (Samalova et al., 2020). For instance, the *ecm33* genes encoding GPI-anchored proteins have been identified in *B. bassiana* (*Bbecm33*) and *M. robertsii* (*Mrecm33*). Disruptions of these genes lead to reduced conidiation and enhanced sensitivity to cell wall-perturbating agents, oxidants and fungicides (Chen et al., 2014). Similarly, in *A. flavus* and *A. fumigatus*, GPI-anchored proteins are required for fungal growth, conidial germination, aflatoxin biosynthesis, and seed colonization (Li et al., 2007; Chang et al., 2018). Hydrophobins, cysteine-rich proteins found in filamentous fungi, are critical for cell wall surface hydrophobicity, adhesion to insect cuticles, UVB resistance, heat tolerance, and virulence in *M. brunneum* and *M. robertsii* (Sevim et al., 2012; Zhang et al., 2023). In *B. bassiana*, two hydrophobins contribute to cell surface hydrophobicity, adhesion, virulence, and the formation of the rodlet layer on the conidial coat (Sevim et al., 2012). Beta-1,3-glucanase plays a critical role in fungal pathogenesis by enhancing invasive capabilities and evading host immune defenses. In *B. bassiana*, the endo-β-1,3-glucanase (BbEng1) is located on the cell membrane, remodeling pathogen-associated molecular patterns on the fungal wall to evade immune detection. This immune evasion mechanism enhances fungal survival in the host. Overexpression of β-1,3-glucanase in *M. robertsii* and *M. acridum* has also been linked to increased pathogenic potential (Wang et al., 2023). The findings in this study highlight the critical role of PKS15 and its associated polyketide in cell wall synthesis, possibly through interactions with GPI-anchored proteins, hydrophobins, or β-1,3 linked cell wall components. This result aligns with previous studies showing impaired cell wall surface structure in the Δ*pks15* mutant (Toopaang et al., 2017; Udompaisarn et al., 2020).

Since PKS15 has been associated with the production of insect-virulent secondary metabolites, including beauvericins, bassianolide, enniatin A, and intracellular siderophore (Toopaang et al., 2023), we conducted DEG analysis of secondary metabolite BGCs in Δ*pks15 in vivo* compared to WT *in vivo*. Using antiSMASH, 45 BGCs were identified. In WT *in vivo* relative to WT *in vitro*, several genes in NRP clusters, T1-PKS clusters, hybrid NRP-T1PKS clusters, fungal RiPP clusters, hybrid NRP-fungal RiPP clusters, and terpene clusters were upregulated. Furthermore, in Δ*pks15 in vivo* relative to WT *in vivo*, these genes were downregulated in Δ*pks15 in vivo* under similar conditions. The identified genes included those encoding UDP-glucosyl transferase (from the *pks15* cluster), arylsulfotransferase, allergen V5/Tpx-1 family protein, nonribosomal peptide synthetase, hypothetical proteins, N,O-diacetyl muramidase, mannitol 1-phosphate dehydrogenase, Tat pathway signal sequence, prolyl oligopeptidase, MFS superfamily transporter, LysM domain-containing protein, hydrophobin, subtilisin, and subtilase. These genes play critical roles in host immune defense, detoxification, conidiation, stress response, insect cuticle penetration and secondary metabolite production.

The UDP-glucosyl transferase (UGT) is essential in several biological processes because it transfers nucleotide sugars (UDP-glucose, UDP-galactose, UDP-xylose, and UDP-rhamnose) to acceptors such as carbohydrates, proteins, and other chemicals for polysaccharides biosynthesis, cell wall formation, and glycosylation of various secondary metabolites (Bowles et al., 2005; Gow et al., 2017; Marschall et al., 2019). In another strain of *B. bassiana* ARSEF2860, the genes associated with cell wall construction and regulation, such as galactomannoprotein and glycosyltransferase, were differently expressed at 72 h after application of conidia on whiteflies (Xia et al., 2013). On the other hand, UGT are the enzymes responsible for the xenobiotic detoxification in filamentous fungi by adding polar molecules to substances or creating water-soluble and nontoxic metabolites (Sang et al., 2018). Some microorganisms can utilize UGT to detoxify the host molecules. For instance, baculovirus-derived ecdysteroid UDP glucosyltransferase (EGT) inactivates the molting hormone, 20-hydroxyecdysone (20E), therefore disrupting insect molting and development, including pupation (Shen et al., 2018). Expression of *egt* in *B. bassiana* reduced 20E levels, suppressed insect immune response, decreased phenoloxidase activity, and increased larval mortality (Mao et al., 2023; Zhu et al., 2024). Bacterial arylsulfate sulfotransferases (ASSTs) contribute to the virulence and detoxification of the phenolic compounds (Kim and Kobashi, 1986; Kim et al., 1986). ASSTs also play a role in the biosynthesis of siderophores in *Edwardsiella tarda* and *asst* mutations impaired virulence (Mathew et al., 2001). In addition, ASSTs are involved in the type III PKS biosynthesis of sulfated caprazamycins (CPZs) through the action of Cpz6, Cpz8, and Cpz4 (Tang et al., 2013). Mannitol-1-phosphate dehydrogenase (MPD) is crucial for mannitol metabolism. It contributes to stress tolerances and pathogenicity in *B. bassiana*, with *MPD*-knockout mutants exhibiting reduced conidial germination, decreased tolerance to oxidative stress, UV-B irradiation and heat, leading to lower infectivity against *Spodoptera litura* larvae (Wang et al., 2012; Garza-López et al., 2015). N,O-diacetyl muramidase, involved in mycoparasitism, facilitates antagonistic interactions between endophytes and pathogens by targeting the cell wall (Waqar et al., 2023), as seen in *T. atroviride*, when co-cultured with the pathogenic fungus *Guignardia citricarpa* (de Lima et al., 2016). MFS transporters, essential for nutrient uptake and toxin secretion (Perlin et al., 2014; Lai et al., 2017), were upregulated in entomopathogenic fungi *M. acridum* and *B. bassiana*, facilitating the transport of siderophores and penicillin (Law et al., 2008; Yang et al., 2012; Wang et al., 2016; Park et al., 2023). LysM proteins, crucial virulence factors in *B. bassiana*, shield fungal cell wall chitin from host immune detection and suppress pathogen-associated molecular patterns (PAMPs) to downregulate host immune responses (De Jonge et al., 2010; Cen et al., 2017). Subtilisin and subtilase (Pr1) proteases, which degrade the insect cuticle, were upregulated during appressorium formation, hyphal invasion, and emergence through the cuticle (Leger et al., 1991; Cherrie-Lee and Bidochka, 2005; Gao et al., 2020) and also contribute to nematode and insect egg degradation in nematophagous fungi (Larriba et al., 2012; Lai et al., 2014). Overall, the downregulation of these genes in Δ*pks15* suggests that PKS15 and its associated polyketides play a critical role in fungal virulence, stress adaptation, and host interactions, reinforcing their importance in secondary metabolite biosynthesis and pathogenicity.

**Figure 7:**
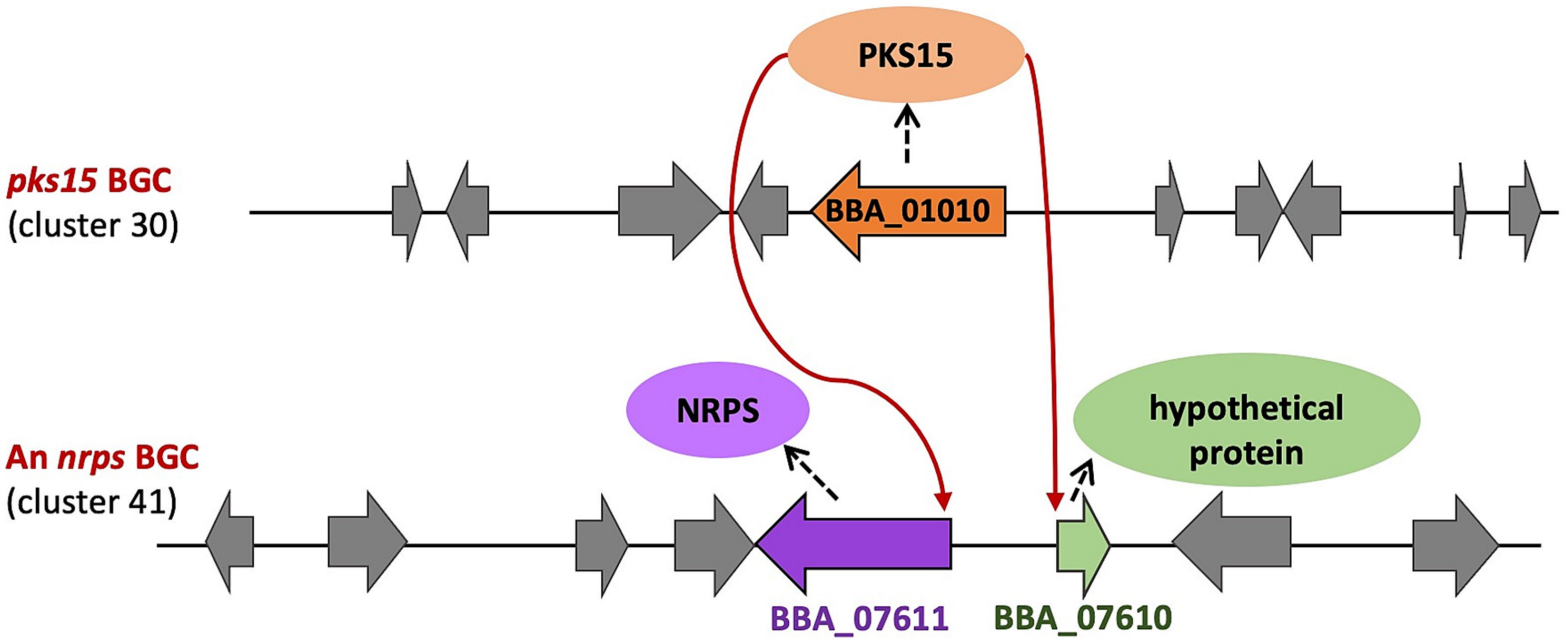
Proposed crosstalk between the *pks15* BGC and NRPS cluster 41. PKS15 or its associated polyketide product may function as a regulator, influencing the expression of the *nrps* gene and a gene encoding a hypothetical protein in cluster 41 (red arrows).

Remarkably, in the NRPS cluster 41, both an NRPS gene of unidentified metabolite (BBA_07611) and a gene encoding a hypothetical protein (BBA_07610) were consistently downregulated in the Δ*pks15 in vivo*. In addition, our study further demonstrated the downregulation of an unidentified functional protein in the bassianolide cluster 44, supporting the previous finding that the promoters of *pks15* and the bassianolide cluster share conserved regulatory motifs. Consistently with the transcriptomic data, NRP production also decreased in Δ*pks15* mutant during host infection (Toopaang et al., 2023). These results suggest that PKS15 or its associated polyketide product may function as a regulator, directly modulating the expression of genes in NRPS clusters, particularly the *nrps* genes, thereby influencing the biosynthesis of secondary metabolites (Figure 7).

Functional crosstalk enhances the structural diversity and bioactivity of secondary metabolites, and interactions between NRPS and PKS BGCs have been demonstrated in the biosynthesis of various bioactive compounds. For instance, in *Penicillium crustosum*, gene-level crosstalk between PKS clusters facilitates the coupling of the clavatol (*cla*) and terrestric acid (*tra*) gene clusters, leading to the biosynthesis of the fungal polyketides penilactones A and B (Fan et al., 2019). Similarly, in *Daldinia eschscholzii* TL01, crosstalk between the chromane (*chr*) and naphthalene (*nap*) gene clusters results in the production of novel immunosuppressive polyketides, such as dalmanol A and acetodalmanol A (Dai et al., 2022). In *A. terreus*, azasperpyranone A is synthesized through the interaction of two BGCs: BGC A, forming the polyhydric phenol, and BGC B constructing the azaphilonoid scaffold (Huang et al., 2020). In addition, in *A. carbonarius*, the PKS gene *Acpks* affects the expression of the global transcription factor gene *laeA*, which regulates ochratoxin A biosynthesis (Maor et al., 2021). Crosstalk also occurs among NRPS clusters, as seen in *A. fumigatus*, where spirotryprostatin production is facilitated by two distinct pathways: the epoxide route catalyzed by FMO FqzB and the radical route catalyzed by cytochrome P450 FtmG (Tsunematsu et al., 2013). Furthermore, echinocandin biosynthesis depends on crosstalk between the *hty* BGC, which generates L-homotyrosine, and the *ecd* BGC, which incorporates it into the final product (Dai et al., 2022). A similar mechanism is observed in *Saccharopolyspora erythraea*, where 2,5-diketopiperazine siderophore biosynthesis involves two different NRPS BGCs: the gene *ercD* in the NRPS5 cluster (responsible for erythrochelin biosynthesis) and the GCN5-like N-acetyltransferase gene *mcd* in the NRPS1 cluster, located 2 Mbp apart (Lazos et al., 2010). Crosstalk between an NRPS cluster and a PKS cluster is also observed in *A. nidulans*, where the expression of the regulatory gene *scpR* in the NRPS gene cluster encoding ScpR resulted in its binding to the promoter of *afoA* in another PKS gene cluster, facilitating the biosynthesis of polyketide asperfuranone (Bergmann et al., 2010). Several key genes identified in this study exhibit functional analogies across diverse pathogenic microorganisms, supporting the concept that *B. bassiana* employs evolutionarily conserved mechanisms, including virulence factor production, cell wall remodeling, and stress responses. Notably, the PKS15-associated regulatory crosstalk uncovered in this study suggests a unique role in transcriptionally coordinating genes across distinct BGCs, thereby contributing to both secondary metabolism and fungal pathogenicity.

In conclusion, our transcriptomic analysis highlights the role of PKS15 as a key regulator that orchestrates fungal cell wall structure and secondary metabolite production at the transcriptional level. To our knowledge, this is the first transcriptomic evidence that the *pks15* cluster coordinated with multiple secondary metabolite clusters, including bassianolide, siderophores, tenellin, oosporein, and several previously uncharacterized PKS and NRPS clusters. These clusters are potentially involved in fungal development and insect pathogenicity. Notably, *pks15* was also interconnected with critical biological pathways related to fungal cell wall architecture, including genes encoding GPI-anchored proteins, hydrophobins, and β-1,3-glucan-associated components, which are essential not only for maintaining cell wall integrity but also against host immune responses. Intriguingly, *pks15* was also associated with the Tat pathway, a well-known virulence factor in bacterial pathogens but largely unexplored in entomopathogenic fungi. Furthermore, our findings were corroborated by our metabolomic data (Toopaang et al., 2023), demonstrating upregulation of virulence-associated metabolites including beauvericin, bassianolide, enniatin, and ferricrocin in the *pks15*-overexpressing strain during host infection, reinforcing the role of *pks15* in regulating secondary metabolites that contribute to the pathogenicity of *B. bassiana*. This discovery extends the functional scope of iterative PKSs beyond biosynthesis, suggesting that a previously unrecognized regulatory network contributes to fungal pathogenicity.

Importantly, the elucidation of PKS15’s regulatory functions emphasizes the critical need to identify the chemical structure of its associated polyketide product. Uncovering the structure–function relationship of PKS15 products will not only deepen our understanding of fungal pathogenic mechanisms but also unlock opportunities to engineer advanced biocontrol strategies. Such targeted manipulation could enhance *B. bassiana*’s biocontrol efficacy and facilitate the development of novel bioactive compounds for agricultural and pharmaceutical applications.

## Data Availability

The original contributions presented in the study are included in the article/Supplementary material, further inquiries can be directed to the corresponding authors.

## Footnotes

^1^ https://www.uniprot.org/id-mapping

^2^ https://fungismash.secondarymetabolites.org/#!/start

^3^ https://version-12-0.string-db.org/cgi/input?sessionId=bg8VFUVePBee&input_page_show_search=on

## Supplementary material

SUPPLEMENTARY TABLE S1 Log_2_ fold changes of upregulated genes in the comparative analysis of WT *in vivo* vs. WT *in vivo* and downregulated Δ*pks15* *in vivo* vs. WT *in vivo* related to mycotoxin biosynthesis.

SUPPLEMENTARY TABLE S2 Log_2_ fold changes of upregulated genes in the comparative analysis of WT *in vivo* vs. WT *in vivo* and downregulated Δ*pks15* *in vivo* vs. WT *in vivo* related to secondary metabolite biosynthesis.

SUPPLEMENTARY TABLE S3 Log_2_ fold changes of upregulated genes in the comparative analysis of WT *in vivo* vs. WT *in vitro* and downregulated Δ*pks15* *in vivo* vs. WT *in vivo* related to cell wall synthesis.

SUPPLEMENTARY TABLE S4 Log_2_ fold changes of expressed genes from different types of secondary metabolite BGCs showing upregulated genes (green) and downregulated genes (grey) in the comparative analysis of WT *in vivo* vs. WT *in vitro* and ∆*pks15* *in vivo* vs. WT *in vivo*.

SUPPLEMENTARY TABLE S5 The influence of *pks15* cluster on the log_2_ fold changes of DEGs in other secondary metabolite BGCs at 48, 72, and 96 h.
